# Supplementary figures and images for: CoPhosK: A method for comprehensive kinase substrate annotation using co-phosphorylation analysis
Source: PLoS Comput Biol. 2019 Feb 27;15(2):e1006678. doi: 10.1371/journal.pcbi.1006678 (PMC6411229; doi:10.1371/journal.pcbi.1006678)

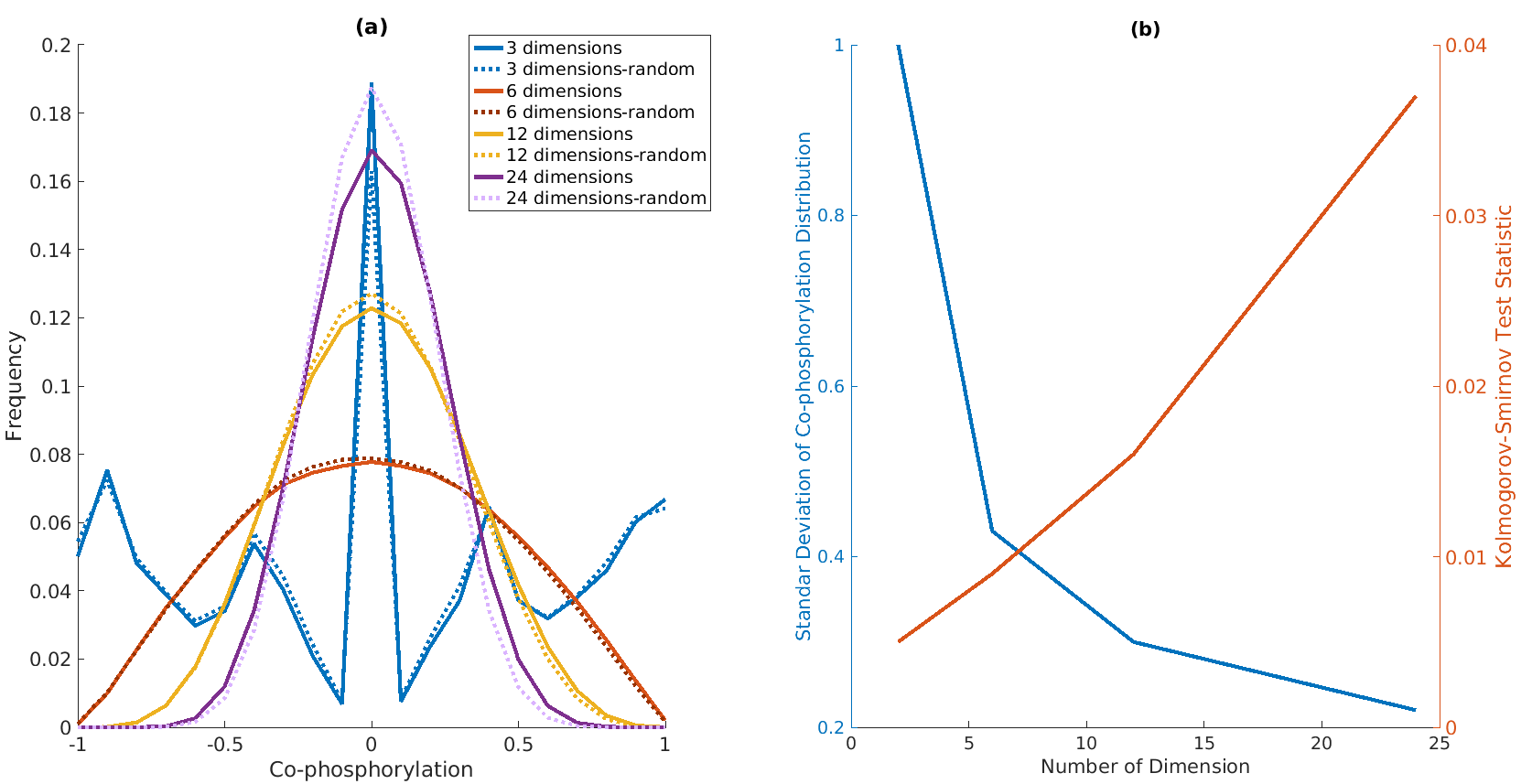

Supplement: S1 Fig — Using the 24 breast cancer PDX samples, (a) we randomly selected subsets of samples and plot the co-phosphorylation distribution among phosphosites (solid line). We also randomize that data to compare against the co-phosphorylation distribution in original data (dashed line). (b) The standard deviation of the co-phosphorylation distribution is shown in the blue line and red line shows changes of Kolmogorov-Smirnov test statistic (the maximum absolute difference between cumulative distribution of co-phosphorylation in original and permuted data) in different number of dimensions. (TIF) [file pcbi.1006678.s001.tif]

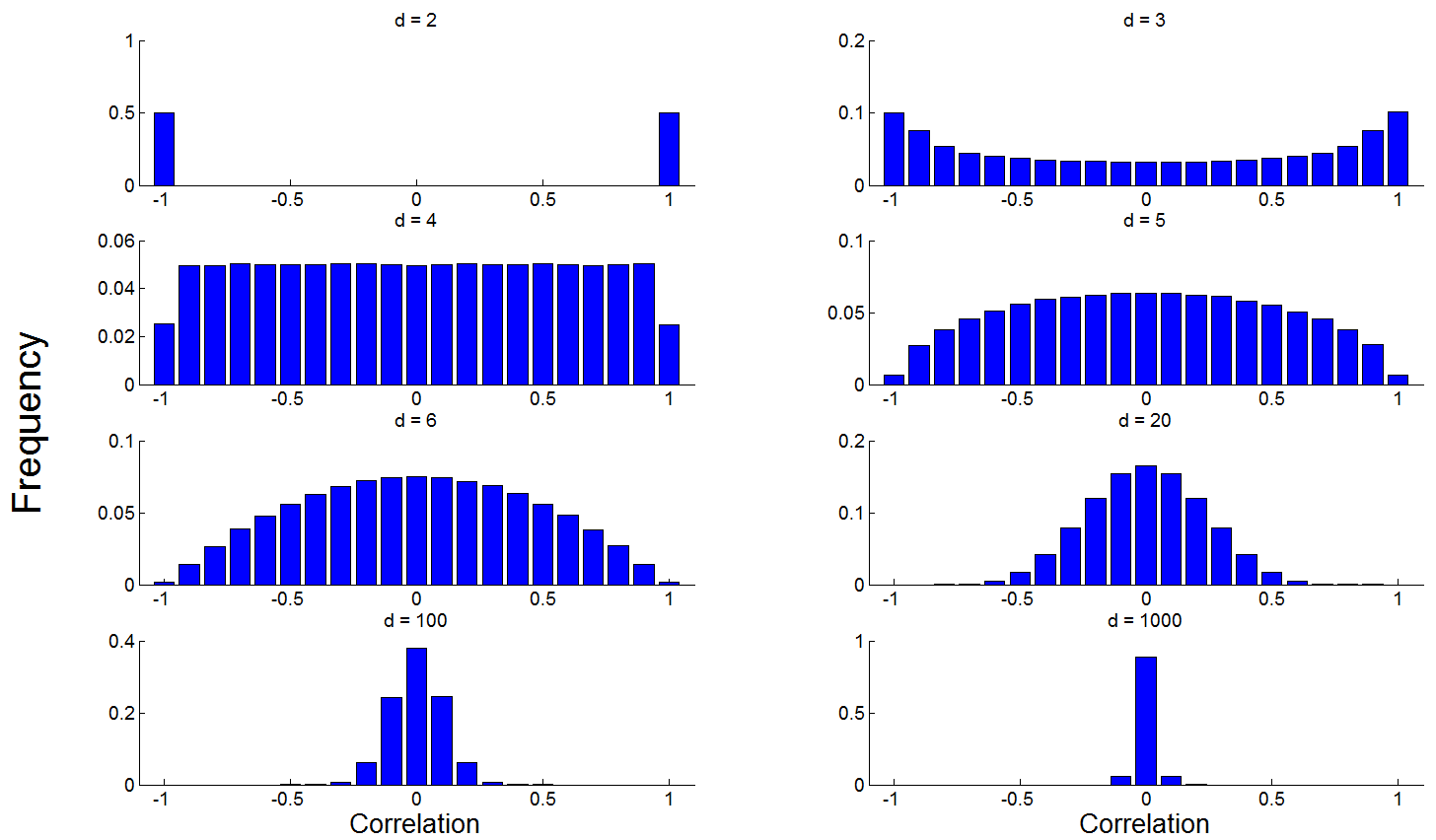

Supplement: S2 Fig — We generated 1000 random vectors from a normal distribution with zero mean and a standard deviation of one and plotted the distribution of correlation among all pairs of vectors. Each panel shows the histogram of correlations among pairs of vectors for a specific number of dimensions (denoted d). (TIF) [file pcbi.1006678.s002.tif]

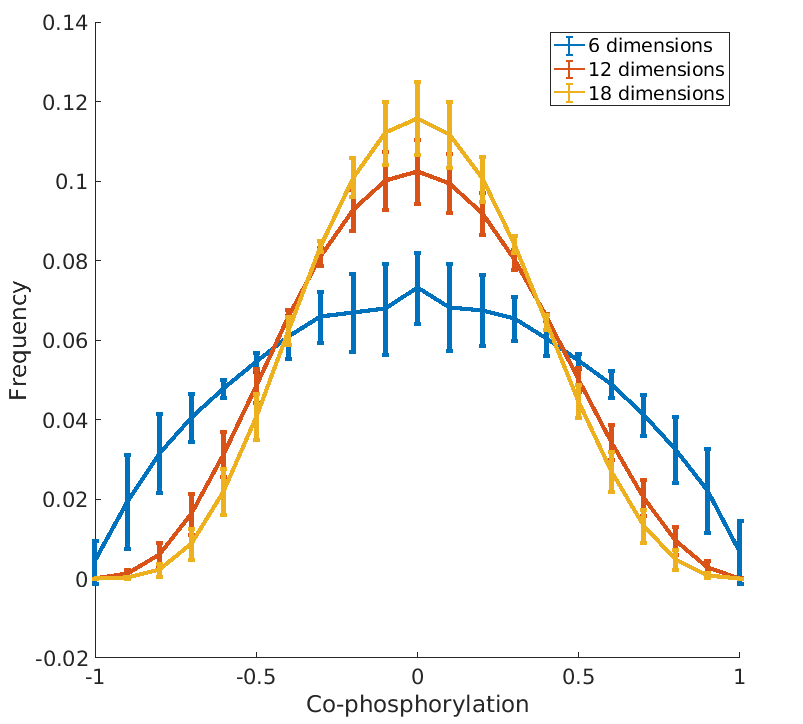

Supplement: S3 Fig — Using the 24 breast cancer PDX samples, we randomly select 6, 12 and 18 subsets of samples 100 times and plot the co-phosphorylation distribution among phosphosites. (TIF) [file pcbi.1006678.s003.tif]

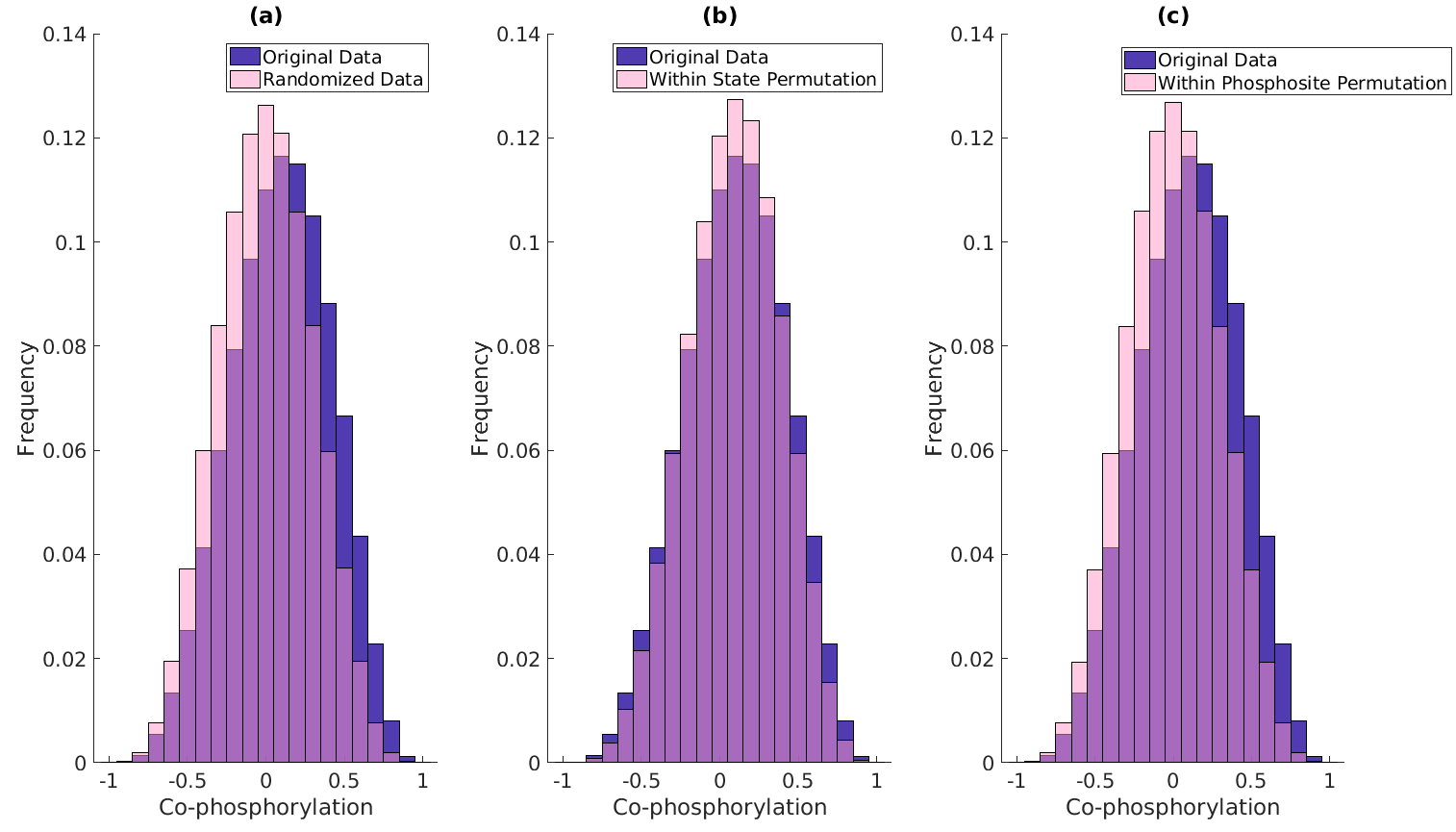

Supplement: S4 Fig — The blue histogram shows the distribution of co-phosphorylation (the correlation between the phosphorylation levels) of all pairs of phosphosites in ovarian cancer (μ = 0.09,σ = 0.31). The pink histogram in each panel shows the distribution of co-phosphorylation of all pairs of phosphosites in 100 permutation tests representing (a) randomization of all entries in the phosphorylation matrix (μ = 1.6E-5,σ = 0.29), (b) permutation of all entries across phosphosites for each state (μ = 0.08,σ = 0.29), and (c) permutation of all entries across states within each phosphosite (μ = 1.3E-4,σ = 0.29). The distribution of co-phosphorylation in the original dataset is significantly different as compared to the distribution of co-phosphorylation in all permutations (Kolmogorov-Smirnov (KS) test p-value << 1E-9). (TIF) [file pcbi.1006678.s004.tif]

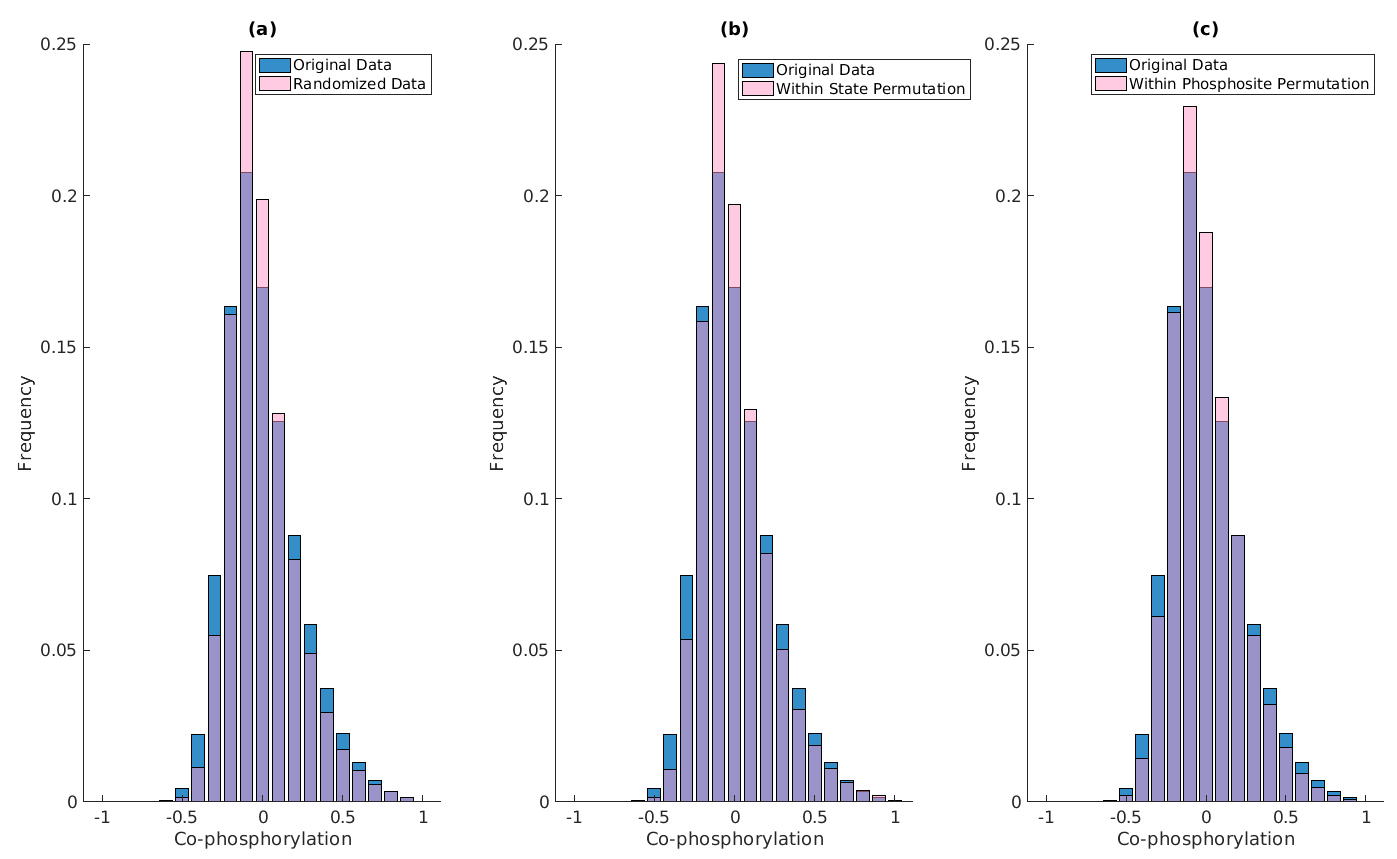

Supplement: S5 Fig — The blue histogram shows the distribution of co-phosphorylation (the correlation between the phosphorylation levels) of all pairs of phosphosites in breast cancer (μ = 0.003,σ = 0.23). The pink histogram in each panel shows the distribution of co-phosphorylation of all pairs of phosphosites in 100 permutation tests representing (a) randomization of all entries in the phosphorylation matrix (μ = -1.6E-5,σ = 0.2), (b) permutation of all entries across phosphosites for each state (μ = 0.005,σ = 0.21), and (c) permutation of all entries across states within each phosphosite (μ = -1.9E-6,σ = 0.2). The distribution of co-phosphorylation in the original dataset is significantly different as compared to the distribution of co-phosphorylation in all permutations (Kolmogorov-Smirnov (KS) test p-value << 1E-9). (TIF) [file pcbi.1006678.s005.tif]

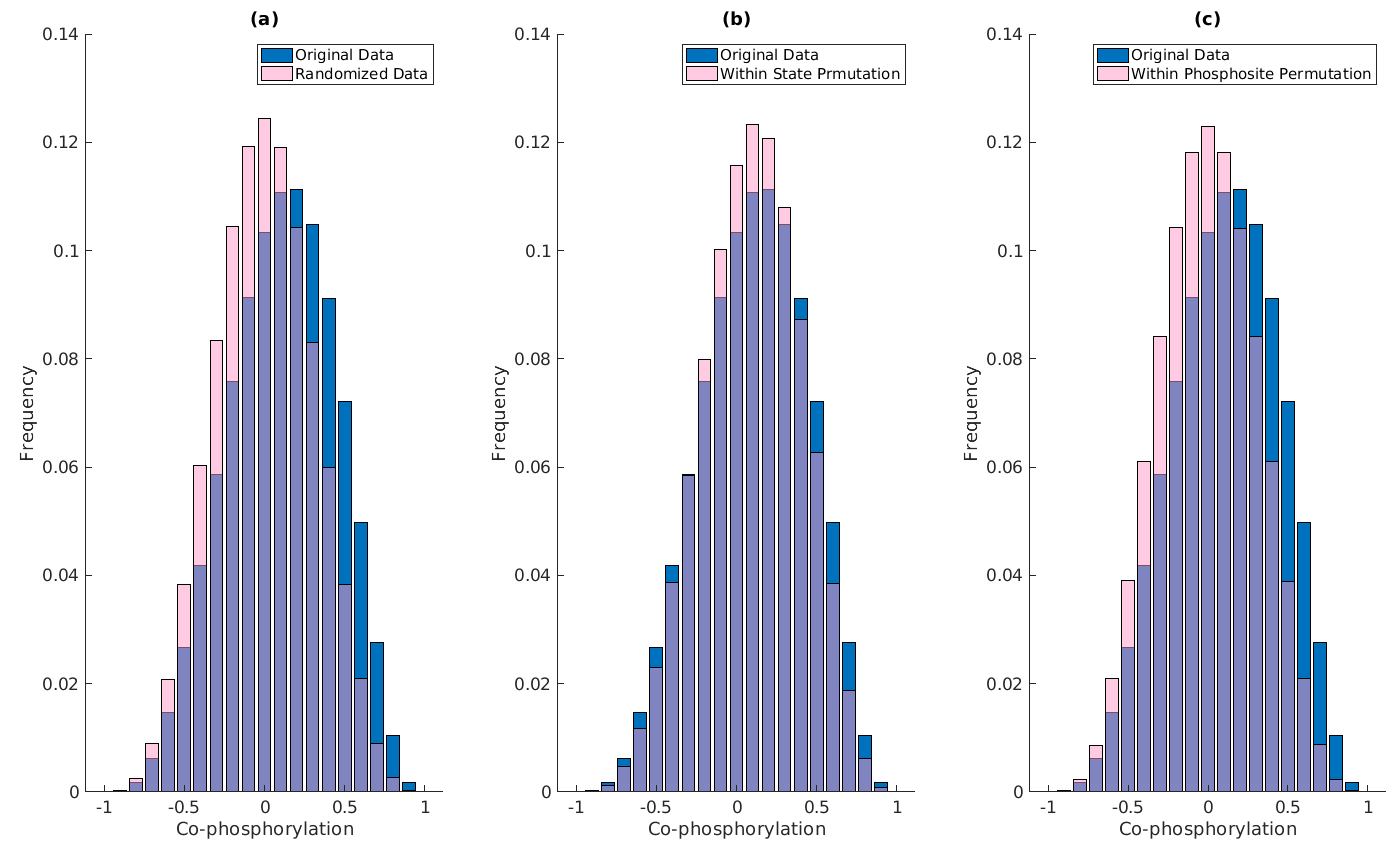

Supplement: S6 Fig — The blue histogram shows the distribution of co-phosphorylation (the correlation between the phosphorylation levels) of all pairs of phosphosites in breast cancer (μ = 0.1,σ = 0.32). The pink histogram in each panel shows the distribution of co-phosphorylation of all pairs of phosphosites in 100 permutation tests representing (a) randomization of all entries in the phosphorylation matrix (μ = -2.6E-5,σ = 0.3), (b) permutation of all entries across phosphosites for each state (μ = 0.09,σ = 0.3), and (c) permutation of all entries across states within each phosphosite (μ = -3.4E-5,σ = 0.3). The distribution of co-phosphorylation in the original dataset is significantly different as compared to the distribution of co-phosphorylation in all permutations (Kolmogorov-Smirnov (KS) test p-value << 1E-9). (TIF) [file pcbi.1006678.s006.tif]

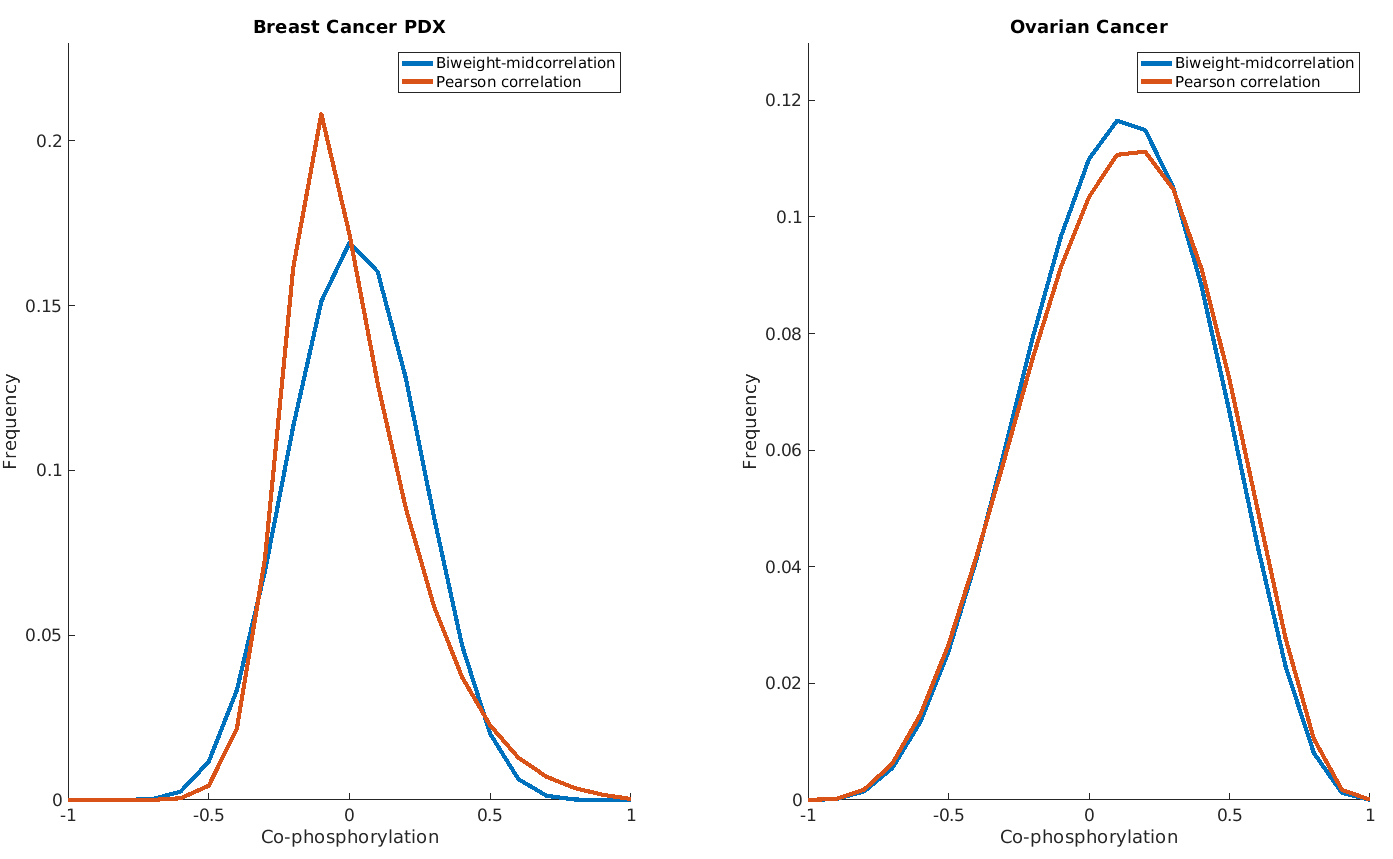

Supplement: S7 Fig — Comparison of co-phosphorylation distribution among phosphosite pairs in (a) breast cancer PDX and (b) ovarian cancer tumors using biweight-midcorrelation (blue curve) and Pearson correlation (red curve). (TIF) [file pcbi.1006678.s007.tif]

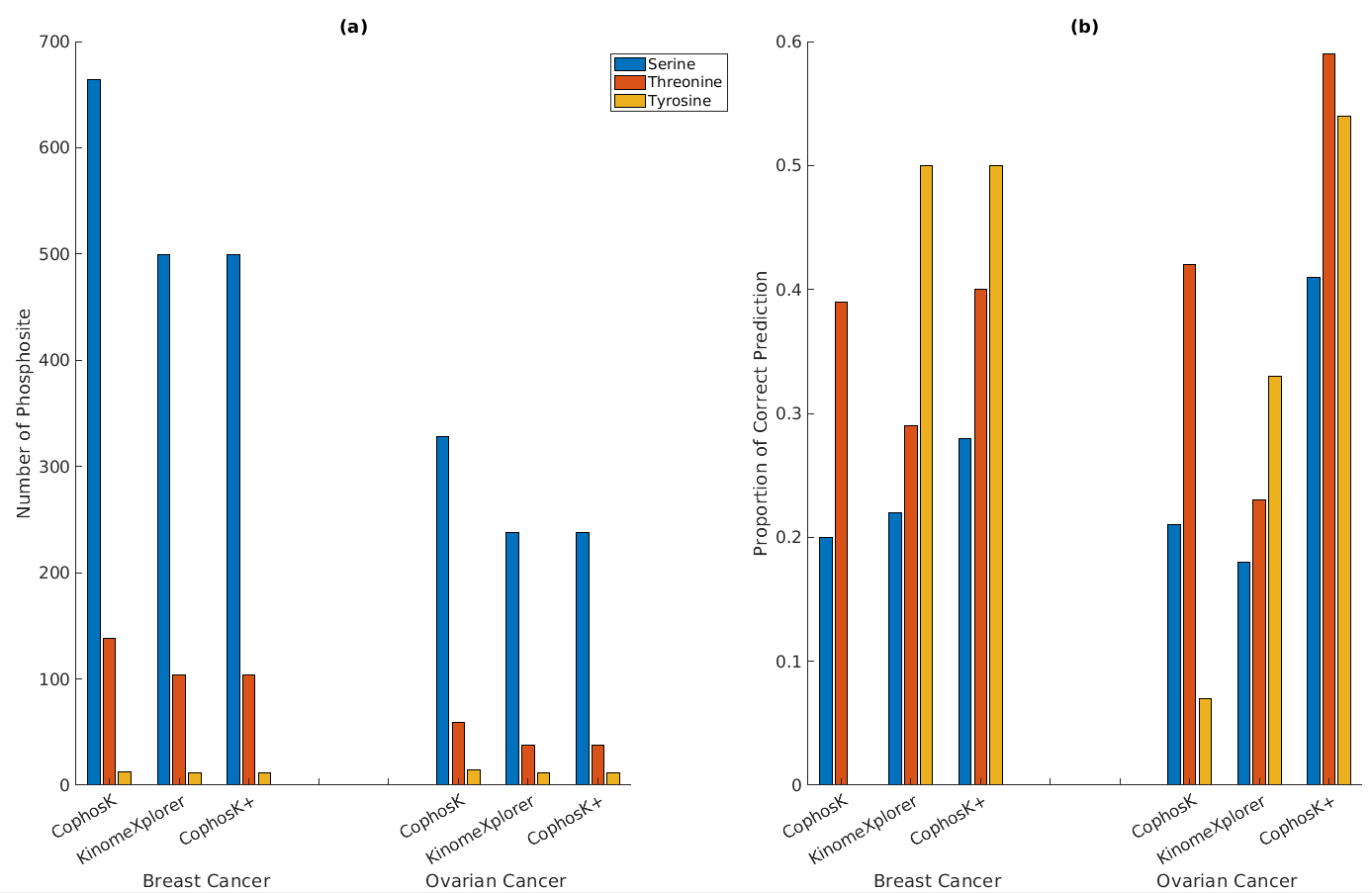

Supplement: S8 Fig — Number of annotated phosphosites and the methods’ performance separated on specific residue is reported in (a) and (b), respectively. (TIF) [file pcbi.1006678.s008.tif]

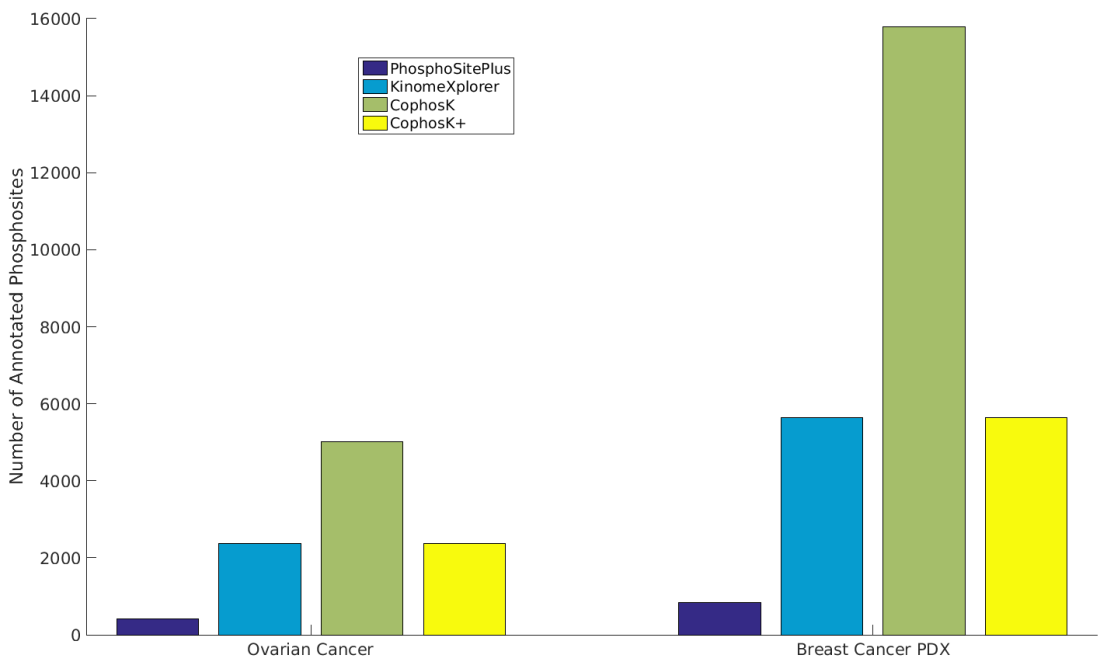

Supplement: S9 Fig — Number of phosphosites in each dataset that are annotated by each method is shown. (TIF) [file pcbi.1006678.s009.tif]

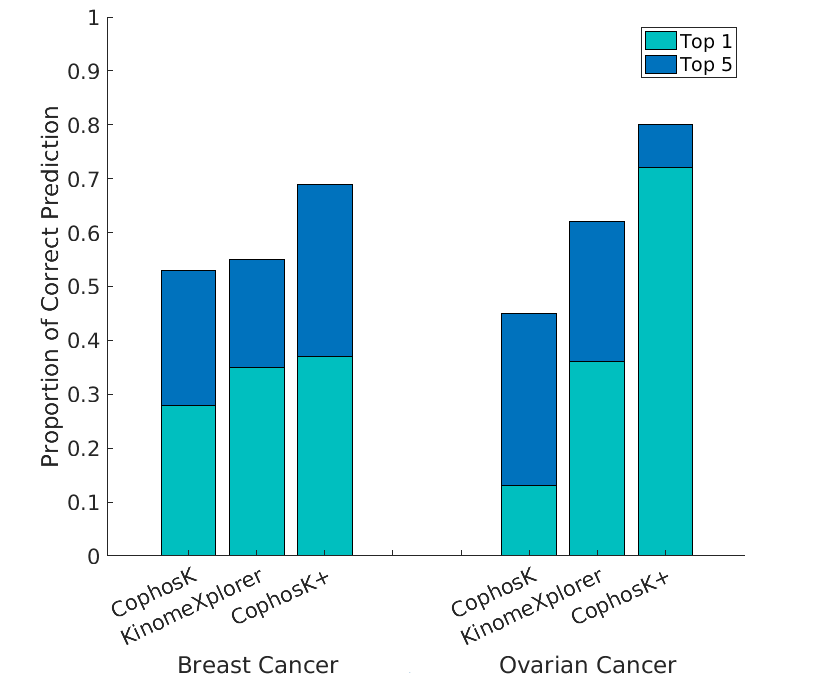

Supplement: S10 Fig — There is 2427 KSAs reported in the Phospho.ELM dataset and 1350 KSAs are common between PhosphoSitePlus and Phospho.ELM (TIF) [file pcbi.1006678.s010.tif]

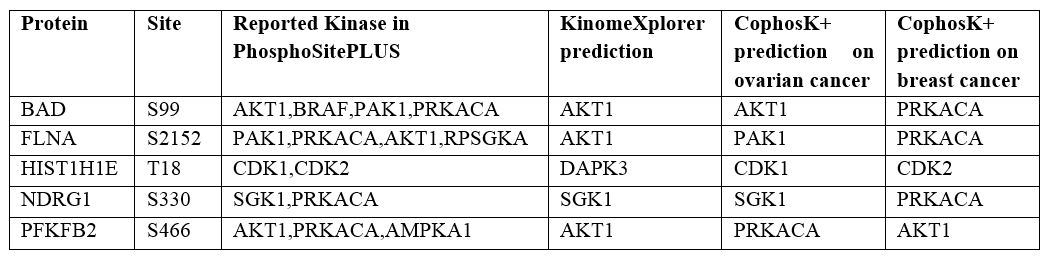

Supplement: S1 Table — The phosphosites listed in this table are reported to have more than one kinase in PhosphoSitePlus. CophosK+ identifies previously reported, but different kinases as the top-ranked candidate based on each dataset. (TIF) [file pcbi.1006678.s011.tif]
